# A systematic review of healthcare-associated infections in Africa: An antimicrobial resistance perspective

**DOI:** 10.4102/ajlm.v7i2.796

**Published:** 2018-12-06

**Authors:** Emmanuel O. Irek, Adewale A. Amupitan, Temitope O. Obadare, Aaron O. Aboderin

**Affiliations:** 1 Department of Medical Microbiology and Parasitology, Obafemi Awolowo University Teaching Hospitals Complex, Ile-Ife, Osun, Nigeria; 2 Department of Medical Microbiology and Parasitology, Obafemi Awolowo University, Ile-Ife, Osun, Nigeria

## Abstract

**Background:**

Healthcare-associated infection (HCAI) is a global health challenge, not only as an issue of patient safety but also as a major driver of antimicrobial resistance (AMR). It is a major cause of morbidity and mortality with economic consequences.

**Objective:**

This review provides an update on the occurrence of HCAI, as well as the contribution of emerging AMR on healthcare delivery in Africa.

**Methods:**

We searched PubMed, Cochrane database, African Journals Online and Google Scholar for relevant articles on HCAI in Africa between 2010 and 2017. Preferred reporting items of systematic reviews and meta-analyses guidelines were followed for selection. Thirty-five eligible articles were considered for the qualitative synthesis.

**Results:**

Of the 35 eligible articles, more than half (*n* = 21, 60%) were from East Africa. *Klebsiella* spp., *Staphylococcus aureus, Escherichia coli* and *Pseudomonas* spp. were the common pathogens reported in bloodstream infection, (catheter-associated) urinary tract infection, surgical site infection and healthcare-associated pneumonia. Among these various subtypes of HCAI, methicillin-resistant *S. aureus* (3.9% – 56.8%) and extended-spectrum beta-lactamase producing Gram-negative bacilli (1.9% – 53.0%) were the most reported antimicrobial resistant pathogens.

**Conclusion:**

This review shows a paucity of HCAI surveillance in Africa and an emergence of AMR priority pathogens. Hence, there is a need for a coordinated national and regional surveillance of both HCAI and AMR in Africa.

## Background

Healthcare-associated infection (HCAI) is a global health challenge, not only as an issue of patient safety but also as a major driver of antimicrobial resistance (AMR). The emergence and spread of AMR threatens effective control and treatment of various infections worldwide.^1,2^ These infections, often caused by multidrug-resistant organisms, take a heavy toll on patients and their families by causing illness, prolonged hospital stay, potential disability, excess costs and sometimes death.^3,4,5^ Thus, HCAIs are major causes of preventable morbidity and mortality in low- and middle-income countries where infection rates are relatively higher due to poor infection control practices, inappropriate use of limited resources, under-staffing of healthcare facilities and overcrowding of hospitals.^6^

Healthcare-associated infections rank among the 10 leading causes of death in the United States, accounting for 1.7 million affected individuals and about 99 000 deaths in 2002 and resulting in up to USD33 billion of excess medical costs every year.^7^ In England, more than 100 000 cases of HCAI are estimated to cost £1 billion and directly cause over 5000 deaths annually.^5^ Although data are sparse, evidence suggests HCAI exerts greater burden in developing countries. Pooled prevalence of HCAI in developing countries is 15.5 per 100 patients (95% confidence interval; 12.6–18.9), with surgical site infections being the leading HCAI, caused mainly by Gram-negative organisms and multidrug-resistant organisms. Multidrug-resistant organisms account for 25% of HCAI globally.^2,5^

There are different types of HCAIs as highlighted by the National Healthcare Safety Network patient safety component manual of the United States Centers for Disease Control and Prevention.^8,9^ These include urinary tract infection (UTI), which is usually catheter-related, surgical site infection (SSI), bloodstream infection (BSI) – laboratory-confirmed bloodstream infection or central-line associated bloodstream infection – and pneumonia (clinically-defined pneumonia or ventilator-associated). Other HCAIs occur in the bones, joints, central nervous system, cardiovascular system (e.g. endocarditis) and in the skin and soft tissue.

Generally, HCAI pervades all health systems across the divide of developed and developing economies globally. For every 100 hospitalised patients at any given time, 7 in developed and 10 or more in developing countries will acquire at least one HCAI.^7^ Moreover, about 5% – 10% of patients admitted to hospitals in developed countries acquire one or more HCAIs, with 15% – 40% of those admitted into the intensive care unit being most affected.^2,5^ Antimicrobial resistant pathogens involved in HCAI include methicillin-resistant *Staphylococcus aureus* (MRSA), penicillin-resistant pneumococci, vancomycin-resistant enterococci, extended-spectrum beta-lactamase (ESBL)-producing Enterobacteriaceae and carbapenem-resistant Enterobacteriaceae.

The epidemiological gaps leading to the absence of reliable estimates of the global burden are mainly because surveillance of HCAI consumes time and resources and requires expertise in data collection, analysis and interpretation.^5,10^ Previous systematic reviews of HCAI in developing countries^11^ covered the period between 1995 and 2008, while another, which focused on the World Health Organisation (WHO) African region, covered between 1995 and 2009.^12^ These reviews highlighted the need for boosting microbiological diagnostic capacity for HCAI, increased infection prevention and control (IPC) practices, as well as frequent surveillance of HCAI. Only a few African countries have established national surveillance systems for HCAI as emphasised by the WHO patient safety module.^10^ Furthermore, pockets of data on HCAI from different healthcare facilities across Africa differ in methodological approach. This review provides an update (2010–2017) on the occurrence of HCAI, as well as the contribution of emerging antimicrobial resistance in healthcare delivery in Africa.

## Systematic search methods

**FIGURE 1:**
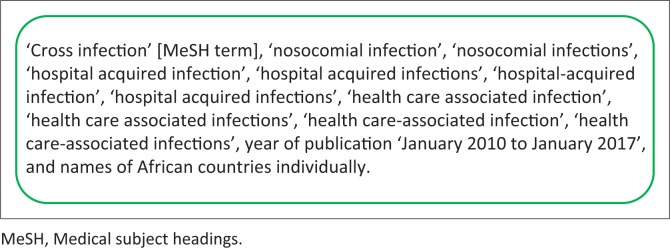
Search terms used in the systematic review.

**FIGURE 2:**
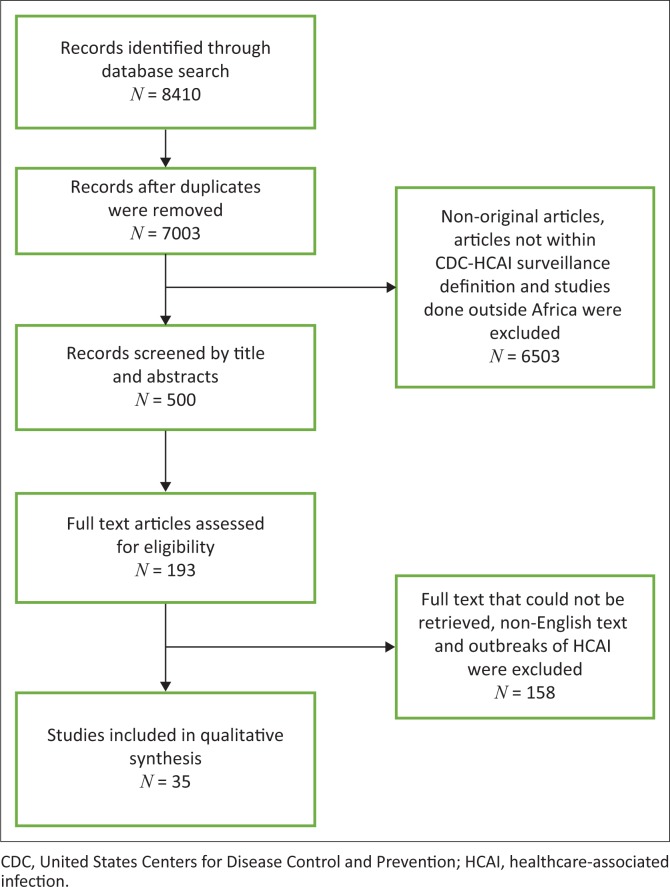
Summary of article selection.

This systematic review was conducted in line with the Preferred Reporting Items for Systematic reviews and Meta-Analyses (PRISMA), guidelines.^13^ PubMed, Cochrane database, and African Journals Online databases were primarily searched for relevant articles using specific search terms (Figure 1). Other articles were obtained from Google Scholar. The literature search included articles from January 2010 to January 2017. The review included articles written only in the English language, as well as articles on all types of patient populations. We excluded duplicate articles, publications reporting the same data, outbreaks of HCAI and data of surveillance beyond Africa. We obtained the full text of potentially relevant studies and scrutinised them independently. Then we screened the potentially relevant studies for further eligibility Figure 2.

Criteria for selecting the articles included definitions used for HCAI diagnosis, reported HCAI prevalence or incidence, identified microbiological isolates and patterns of antimicrobial resistance (when documented). We only judged microbiological data suitable for assessment when the number of bacterial isolates was reported in relation to in-patients having suspected HCAI. Healthcare-associated infections included in this review were as defined by the United States Centers for Disease Control and Prevention,^9^ that is, infections that develop in in-patients on or after the third (> 48 h) day of admission. Hence, we identified catheter-associated BSI, SSI, UTI (catheter-associated or not), pneumonia (ventilator-associated or not) as the major subtypes of HCAI, and we categorised other infections associated with healthcare service delivery, such as gastroenteritis, skin and soft tissue infection, as ‘others’.

## Literature search and characteristics of the studies included in the systematic review

We identified 8410 records from the search of the electronic databases. The number of full text articles screened was 7003 after the removal of duplicate studies, of which 193 studies were potentially eligible. However, only 35 articles were finally selected for qualitative synthesis for this review according to the aforementioned inclusion criteria.^9,11^ Data were pooled from both prevalence and incidence studies and afterwards summarised in Table 1. The prevalence of infection refers to infected patients per patients present in the hospital or ward at a given point in time.

**TABLE 1:** Summary of eligible reviewed articles on healthcare-associated infection in Africa published between 2010 and 2017.

No.	Country	Occurrence [*n* (%)]					Common organisms isolated					AMR	Ref
			
		BSI	SSI	(CA) UTI	(VA)P	Others	BSI	SSI	(CA)UTI	(VA)P	Others		
1	Egypt	82 (92)	-	-	7 (8)	-	*Klebsiella* spp. *Staphylococcus aureus* *Pseudomonas* spp. *Escherichia coli*	-	-	*Klebsiella* spp. *S. aureus* *Pseudomonas* spp. *E. coli*	-	NR	^28^
2	Egypt	-	-	-	73 (51)	-	-	-	-	*K. pneumoniae P. aeruginosa* *E. coli* *Fungi*	-	ANS	^20^
3	Egypt	12 (23)	-	12 (23)	12 (23)	-	*Enterococcus faecium*	-	*E. faecium*	*E. faecium*	-	VREF	^15^
4	Egypt	154 (33.3)	27 (5.8)	152 (32.9)	90 (19.5)	-	*S. aureus* *Klebsiella* spp.*Pseudomonas* spp. *E. coli*	*S. aureus* *Klebsiella* spp. *Pseudomonas* spp. *E. coli*	Candida spp. *Klebsiella* spp.*Pseudomonas* spp. *E. coli*	*Pseudomonas* spp. *Klebsiella* spp. *S. aureus Proteus* spp.	-	MRSA	^29^
5	Ethiopia	-	138 (71.1)	-	-	-	-	*S. aureus* *Klebsiella* spp. *E. coli* *Proteus* spp. *P. aeruginosa*	-	-	-	NR	^21^
6	Ethiopia	19 (14.1)	69 (51.1)	9 (6.7)	25 (18.5)	GIT 5 (3.7) SST 5 (3.7)		*Klebsiella* spp. *S. aureus* *P. aeruginosa* *E. coli*	*Klebsiella* spp. *S. aureus* *P. aeruginosa* *E. coli*	*Klebsiella* spp. *S. aureus P. aeruginosa* *E. coli*	*Klebsiella* spp. *S. aureus* *P. aeruginosa* *E. coli*	NR	^30^
7	Ethiopia	-	73 (39.7)	-	-	-	-	*S. aureus*	-	-	-	MRSA	^16^
8	Ethiopia	30 (10.2)	7 (2.4)	-	-	-	*S. aureus* CoNS *E. coli* *P. aeruginosa*	*S. aureus* CoNS *E. coli* *P. aeruginosa*	-	-	-	ANS	^42^
9	Ethiopia	-	-	36 (49.32)	-	-	-	-	*P. aeruginosa*	-	-	NR	^45^
10	Ethiopia	-	96 (75)	-	-	-	-	*S. aureus* *Klebsiella* spp. CoNS *Proteus* spp.	-	-	-	NR	^46^
11	Ethiopia	21 (42)	-	-	-	SST 39 (78)	CoNS *S. aureus* *P. aeruginosa* *K. pneumoniae*	-	-	-	CoNS *S. aureus* *P. aeruginosa* *K. pneumoniae*	NR	^49^
12	Ethiopia	-	-	-	-	SST 95 (83.3)	-	-	-	-	S. aureus	ANS	^17^
13	Ethiopia	-	-	28 (52.8)	-	-	-	-	*Citrobacter* spp. *E. coli* *Enterobacter* spp. *S. aureus*	-	-	NR	^47^
14	Ethiopia	-	20 (19.1)	-	-	-	-	-	-	-	-	NR	^37^
15	Ethiopia	-	90 (84.1)	-	-	-	-	*E. coli* *Acinetobacter* spp. *Klebsiella* spp. *Proteus* spp.	-	-	-	ANS	^43^
16	Ethiopia	-	26 (6.8)	-	-	-	-	-	-	-	-	NR	^38^
17	Ethiopia	-	88 (11.4)	-	-	-	-	-	-	-	-	NR	^39^
18	Gabon	9 (20)	20 (44)	12 (26)	-	SST &, GIT 5 (11)	*S. aureus* *E. coli* *K. pneumoniae* *Streptococcus pyogenes*	*S. aureus* *E. coli* *Enterococcus* ssp. Anaerobes	*E. coli* *Enterococcus* spp. *K. pneumoniae* *Acinetobacter* spp.	-	-	ESBL	^31^
19	Kenya	-	12 (7)	-	-	-	-	*S. aureus* CoNS *P. aeruginosa* *Kluyvera* spp.	-	-	-	NR	^48^
20	Kenya	-	52 (80)	-	-	-	-	*Pseudomonas* spp. *Klebsiella* spp. *P. mirabilis* *E. coli*	-	-	-	NR	^22^
21	Libya	430 (51.8)	-	-	-	-	*S. aureus* *Pseudomonas* spp. *Klebsiella* spp. CoNS	-	-	-	-	MRSA MRCoNS ESBL	^23^
22	Libya	8 (10)	-	15 (19)	31 (39)	-	*Klebsiella* spp. *A. baumannii* *Proteus* spp.	-	*Klebsiella* spp. *A. baumannii* *P. aeruginosa* *Enterobacter cloacae*	*K. pneumoniae* *A. baumannii* *P. aeroginosa* *E. cloacae*	-	ESBL	^32^
23	Nigeria	22 (49)	-	16 (35.6)	-	SST 4 (8.9)	*S. aureus* *E. coli* *K. pneumonia*e *P. mirabillis*	-	*Candida* spp. S. marcescens CoNS *C. albicans*	-	*P. flourescens* *P. mirabilis* *P. aeruginosa* *S. aureus*	MRSA MRCoNS VRE	^33^
24	Nigeria	20 (16.9)	32 (27.1)	39 (33.1)	-	GIT 18 (15.3)	*Enterococcus* spp.	*Enterococcus* spp.	*Enterococcus* spp.	-	*Enterococcus* spp.	VRE	^18^
25	Rwanda	-	88 (3.95)	15 (0.67)	12 (0.54)	-	-	-	-	-	-	NR	^40^
26	South Africa	5.9/1000	-	-	-	-	CoNS *E. faecalis* *K. pneumoniae* *A. baumannii* *C. albicans*	-	-	-	-	NR	^14^
27	South Africa	543 (73.4)	-	-	-	-	*E. coli* *K. pneumoniae* *A. baumannii* complex *S. aureus*	-	-	-	-	ESBL	^44^
28	Tanzania	-	42 (35.6)	-	-	-	-	-	-	-	-	NR	^24^
29	Tanzania	-	78 (14.6)	-	-	-	-	*P. aeruginosa*	-	-	-	NR	^19^
30	Tanzania	-	150 (19.9)	-	-	-	-	*S. aureus* CoNS *P. aeruginosa* *A. baumannii* *K. pneumoniae*	-	-	-	MRSA ESBL	^25^
31	Tunisia	46 (68.7)	-	9 (13.4)	36 (53.7)	-	-	-	-	-	-	NR	^34^
32	Tunisia	-	-	-	-	SST 5 (13.4)	-	-	-	-	-	NR	^35^
33	Tunisia	-	-	51 (18.5)	-	-	-	-	*K. pneumoniae* *E. coli* *E. cloacae* *K. oxytoca*	-	-	ESBL	^26^
34	Uganda	-	216 (68.8)	-	-	-	-	*E. coli* *Klebsiella* spp.*Acinetobacter* spp. *S. aureus*	-	-	-	MRSA ESBL	^27^
35	Uganda	-	4 (21.9)	7 (38)	-	-	-	-	-	-	-	NR	^41^

AMR, antimicrobial resistance; ANS, available but not specified; BSI, bloodstream infection; CoNS, coagulase negative staphylococci; ESBL, extended-spectrum beta-lactamases; GIT, gastrointestinal tract infection; MRSA, methicillin-resistant *Staphylococcus aureus*; MRCoNS, methicillin-resistant coagulase negative staphylococci; NR, not reported; SSI, surgical site infection; SST, skin and soft tissue infection; (CA)UTI, (catheter-associated)urinary tract infection; VAP, ventilator-associated pneumonia VRE, vancomycin-resistant enterococci; VREF, vancomycin-resistant *Enterococcus faecium*.

## Results

**FIGURE 3:**
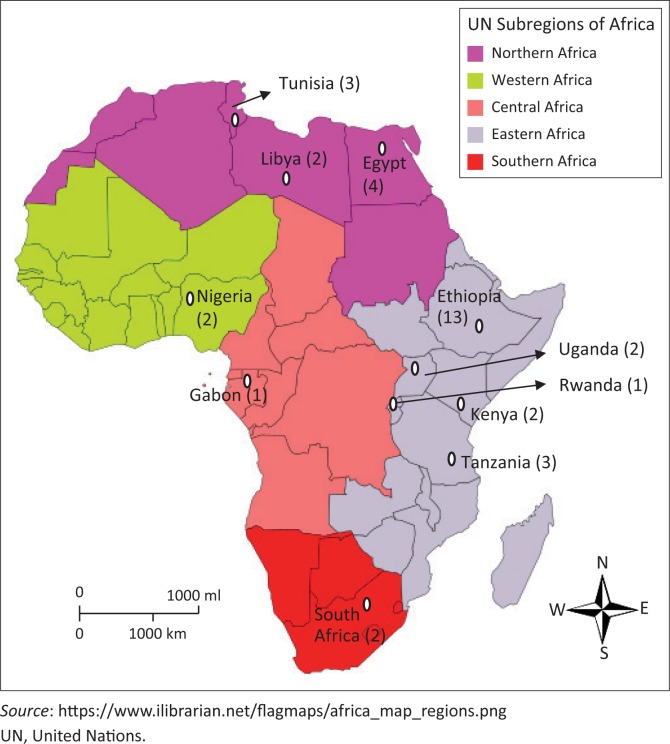
Distribution and number of eligible published articles on healthcare-associated infections in different African countries.

Two-thirds of the reviewed articles were from PubMed, one-third were from African Journals Online and Google Scholar, and none were retrieved from the Cochrane library. Further, more than half (*n* = 21, 60%) of the synthesised articles were from East Africa, whereas the rest were shared across northern, western, southern and central Africa (Figure 3). Only one article reported an incidence study,^14^ whereas the rest were prevalence studies (retrospective or prospective). Five (14.3%) of the reviewed articles based their categorisation on specific microorganisms isolated,^15,16,17,18,19^ whereas some others were based on specific HCAI.^14,16,17,20,21,22,23,24,25,26,35,37,38,39,44,45,46,47,48^ Only eight studies (22.9%)^28,29,30,31,32,33,34,35^ covered HCAI in entirety and conducted full surveillance of the different types enumerated by previous published protocols.^8,36^ Eight articles (22.9%), however, did perform HCAI surveillance without the mention of the microorganisms implicated.^24,34,35,37,38,39,40,41^ The identification of antimicrobial resistance in the panel of laboratory investigation was included in less than half (*n* = 16, 46%) of all the reviewed articles^14,15,17,18,20,23,25,26,27,29,31,32,33,42,43,44^ with only four articles broadly identifying AMR as multidrug-resistant organisms without further characterisation.^17,20,42,43^ In addition, only three of the articles specified the prevailing microorganisms of the various subtypes of HCAI in surveillance.^31,32,33^ The phenotypic method was mostly utilised for the identification of the microorganisms in the laboratory according to Clinical and Laboratory Standards Institute guidelines.^15,16,19,22,23,25,27,31,41,42,43,44,45,46,47,48^

Furthermore, surveillance on (central line-associated) BSI was recorded in 14 (40%) of the reviewed articles^14,15,18,23,28,29,30,31,32,33,34,44,49^ and these were confirmed with blood culture. Some of these articles, however, evaluated only BSI,^14,23,34,44^ whereas others included BSI with other HCAI surveillance subtypes (Table 1). BSI in some individuals was episodic and some others were central line-associated. Diverse microorganisms implicated in BSI in order of decreasing frequency included *Klebsiella* spp., *S. aureus, E. coli, Pseudomonas* spp. and *Acinetobacter* spp. (Table 1). *Klebsiella* spp. and *Staphyloccocus* spp. were the most frequently identified causes of BSI. ESBL producers and methicillin-resistant *Staphylococcus* spp. were the most identified antibiotic-resistant microorganisms in the BSI articles (that mentioned AMR within their panel of laboratory investigation).^23,29,31,32,33,44^ Only one article reported vancomycin-resistant enterococci in BSI,^18^ and another recorded an escalating antibiotic resistance of *Acinetobacter baumannii* to the carbapenems.^32^

Surveillance for SSI was common among the reviewed articles. Over half of the reviewed articles had SSI within the context of their surveillance, of which only 13 focused solely on SSI.^16,19,21,22,24,25,27,37,38,39,43,46,48^ Wound swabs and wound biopsies were specimens taken for microbiological investigation. Some occurrence of SSI were associated with caesarean sections or orthopaedic manoeuvres. The microorganisms most commonly isolated were *S. aureus, E. coli, Klebsiella* spp., *Pseudomonas* spp., in the order of decreasing frequency. Common antimicrobial resistant organisms identified in SSI articles reviewed were MRSA and ESBL-producing Gram-negatives.

In this systematic review, catheter-associated UTI was seen mainly in urologic conditions such as prostatic enlargement and post-gynaecological procedures. Some reviewed articles (*n* = 10; 29%) categorised healthcare-associated UTI (catheter-associated inclusive) as a subset of other HCAI surveillance types.^15,18,29,30,31,32,33,34,40,41^ Common microorganisms isolated from healthcare-associated UTI included *Klebsiella* spp., *E. coli, Enterococcus* spp., and *Pseudomonas* spp. In addition, MRSA, vancomycin-resistant enterococci and ESBL-producing Gram-negative bacilli were the most common antimicrobial resistant pathogens noticed in some identified bacteria for healthcare-associated UTI among the reviewed articles.

Only one study included healthcare-associated pneumonia as a lone subtype of HCAI,^20^ whereas many others included it as a subset of HCAI surveillance types.^15,28,29,30,32,35,40^ Common microorganisms reported among these articles included *Klebsiella* spp., *Pseudomonas* spp., *S. aureus* and *E. coli.* As with other HCAI subtypes in this review, MRSA and ESBL-producing Gram-negative bacilli were the most common antimicrobial resistant pathogens seen. Other HCAI studied in this systematic review were gastroenteritis,^18,30,31^ and skin and soft tissue infection.^17,30,31,33,35,49^

Overall, for the reviewed articles that identified AMR, the prevalence of methicillin-resistant *Staphylococcus* spp. ranged between 3.9% and 80% among the *Staphylococcus* spp. (*S. aureus* and coagulase negative staphylococci) reported.^16,23,25,27,29,33^ The prevalence of Gram-negative bacteria producing ESBL^23,25,26,27,31,32,44^ ranged between 1.9% and 53%, whereas vancomycin-resistant enterococci^15,18,33^ was between 2.54% and 100%.

## Discussion

Until recently in Africa, evidence on the enormity and debilitating effects of HCAI on patients (and relatives of patients) has been low. The resultant effect of many studies conducted in developed countries was to propose a singular surveillance platform for HCAI across their sub-regions.^8,50^ This was intended to identify gaps and target control of HCAI. However, the gravity of HCAI is yet to be fully understood in Africa due to the enormous resource requirements for surveillance and diagnoses.^51^ This was evident by the paucity of studies identified in this review (Figure 3).

Although a well-documented protocol for HCAI has been proffered by the United States Centres for Disease Control and Prevention,^8,9^ only a few studies we reviewed adhered to it or any other protocol of interest. Also, the robustness, reproducibility and inferences from methodology used in the reviewed articles differed considerably from study to study, thus limiting comparability and robust analysis. This systematic review was also limited with search only done in English. Additionally, a follow-up for trends on the HCAI surveillance was rarely conducted in the different healthcare facilities where these studies were conducted. This would have given a clue to either the reduction or the increment of HCAI in such centres, as seen in the archival documentation of the United States CDC.

The prevalent bacteria identified in BSI in this review were *Klebsiella* spp., *S. aureus, E. coli, Acinetobacter* spp., in order of decreasing frequency, which slightly contradicts the order of occurrence in a previous review conducted in South East Asia,^52^ where *Acinetobacter* spp. was found to be the most prevalent organism causing BSI. A similar frequency of identification was seen in SSI surveillance, with *S. aureus* being the most common across reviews with different ecologies but similar healthcare issues of poor funding.^11,52^ Again, as with BSI, *Klebsiella* spp. was the most commonly identified pathogen in this review, which concurs with a similar review by Ling et al.^52^ Although few studies were identified in this review for ventilator-associated pneumonia, *Klebsiella* spp. still remained highly prevalent among other bacteria identified, as noted in a similar study.^52^ These similarities in the bacteria isolated may be due to the likened levels of IPC practices and antibiotic usage, which can influence bacterial fitness.^53^

In addition, the range of occurrence of the AMR patterns in the review articles was quite alarming, considering there have been few or no previous reviews on AMR patterns in HCAI pathogens in Africa. The range of MRSA in this review was higher than that reported in the joint European surveillance of MRSA in HCAI.^54^ This can be adduced to the relatively low IPC practices in Africa,^55^ especially during surgeries or invasive procedures. Also, the occurrence of ESBL-producing Gram-negative bacilli was higher than that obtained by Flokas et al.^56^ that reported 14% in a systematic review of ESBL in paediatric UTIs. Moreover, an increased trend of ESBL has been observed in the United States with recent incidence of about 16.64 infections in 10 000 discharges.^57^

The inadequate IPC strategies instituted in these healthcare facilities to prevent HCAI compromise the quality of healthcare service delivery, hence the prevalence.^11^ Previous reviews on HCAI in developing countries and in the WHO African sub-region^11,12^ have emphasised the need for improved IPC in healthcare facilities to drastically reduce HCAI prevalence. Many of the selected studies^14,20^ mentioned the need for the establishment of IPC, whereas others identified bundle implementation (of the different subtypes of HCAI)^14,20,38,26^ in curbing HCAI in their centres. Only one article studied the aftermath reduction of HCAI using IPC measures.^20^ Thus, studies on interventional IPC measures in the reduction of HCAI are still quite juvenile in Africa. This has been advocated by WHO as a means of measuring and sustaining progress on patient safety.^51^

In this review, most of the reviewed articles highlighted the corresponding AMR patterns of the microorganisms implicated in HCAI,^15,16,18,23,25,26,27,29,31,32,33,44^ whereas others simply mentioned them as multidrug-resistant organisms.^17,20,42,43^ This may be due to inadequate laboratory capacity to identify the specific AMR patterns. This also gives a foreknowledge of the existing prevalence of AMR microorganisms in the healthcare facilities in Africa and a possible spread to the communities if not curtailed. MRSA was identified in a previous review by Allengrazi et al.^11^ as the most prevalent AMR pattern implicated in HCAI. This concurs with the AMR pattern in this review. The presence of ESBL was also noticed to be prevalent alongside MRSA in this review. Carbepenem-resistant organisms have been known globally to cause much mortality and morbidity,^1,58^ and are widely implicated in HCAI,^50,58^ but were rarely mentioned in the synthesised articles. However, one study highlighted carbapenem resistance in *Acinetobacter baumannii* as HCAI in an intensive care unit in Libya.^32^ Furthermore, antimicrobial stewardship has also been identified as a major solution to the rising rates of AMR worldwide.^59,60^ The recognition of this was, however, of little priority in the articles reviewed, with only a limited number of studies^15,18,23,25,27,30,37,31,33^ highlighting the importance of antimicrobial stewardship in the reduction of AMR in HCAI.

Countries in Africa have a wide variation in the capacity to combat AMR in HCAI, but have been greatly hampered by the availability of funds for research, innovation and capacity building.^61,62^ This is worsened by a lower percentage of total health spending in African countries.^63,64^ Thus, the true burden of HCAI in this systematic review is likely to be under-reported and is perhaps greater in countries with weaker health infrastructures. However, this narrative is changing with increasing commitment in Africa to respond to the global threat of AMR. In-country technical capacity with support from partners is now evolving not only to develop national action plans to combat AMR, but also to institute national surveillance for AMR. The WHO Global Antimicrobial Surveillance System provides a tool to standardise data gathering, sharing and analysis through participating institutions and countries at the global level to monitor trends and implement controls.^61,62,65,66^ Moreover, with the current global attention and high–level political commitment to control AMR, funding support for AMR control in Africa is coming from various organisations, which include the WHO, ReAct Africa, the Center for Disease Dynamics, Economics and Policy and the Fleming Fund.^61,67,68^

For sustainability, countries also should have budget lines for AMR control activities either as stand-alone or, more realistically, as part of existing systems such as IPC, maternal and child health and health systems strengthening. Monitoring and evaluation has to be incorporated as the systems develop. Current platforms to do this include the Global Antimicrobial Surveillance System,^66^ which accepts annual surveillance data that have been aggregated in-country, and the Global Point Prevalence Survey,^69^ which monitors antibiotic prescription patterns to enhance stewardship. Hence, report on surveillance and trends of HCAI and AMR occurrence should inform regular updates on guidelines (treatment and IPC) and antibiotic stewardship protocols at the national level, while at the institutional level, evidence will inform accreditation for services or training.

### Conclusion

Although prevention and evolution of HCAI and the reduction of the occurrence of AMR globally have been a primary focus of WHO,^51^ little has been done to combat it in Africa. In addition, surveillance has been known to reduce the burden of HCAI in developed healthcare facilities,^7^ where conscious means of prevention have been instituted accordingly. The inadequate coordination of regional and intra-continental surveillance in Africa led to inconsistencies and non-uniformity in many reported studies of HCAI in this review. This made it difficult to interpret data to display true representativeness. Hopefully, there will be a coordinated national and sub-regional HCAI surveillance as an agenda of the newly created Africa Centres for Disease Control and Prevention.

Finally, this systematic review has compiled all relevant, accessible and eligible studies on HCAI in Africa as a baseline for further insight into developing a concrete surveillance system and strengthening local data collection at healthcare facilities. There seems to be a higher number of studies on HCAI compared to previous reviews.^12^ *Klebsiella* spp. was prevalent across all the HCAI subtypes. MRSA and ESBL-producing Gram-negative bacilli were the notable resistant pathogens identified with worrisome occurrences. These make a strong case for increased laboratory capabilities in the identification of microorganisms and determination of resistance profiles (especially in WHO priority pathogens)^70^ implicated in HCAI. Henceforth, it is desirable that a periodical review of AMR and HCAI in Africa be conducted in view of current interest.
